# Factors associated with positive predictive value of preliminary screening in a two-step screening strategy for colorectal neoplasms in China

**DOI:** 10.1007/s12672-022-00463-8

**Published:** 2022-01-08

**Authors:** Ji-Bin Li, Zhi-Yu Qiu, Yu-Xiang Deng, Yin Li, Zhuo-Chen Lin, Yan-Ping Wu, Fan Weng, Huan Tian, Qing-Jian Ou, Cheng-Hua Gong, Zhi-Zhong Pan, De-Sen Wan, Jian-Hong Peng, Yu-Jing Fang

**Affiliations:** 1 Sun Yat-sen University Cancer Center, Guangzhou, 510060 People’s Republic of China; 2 State Key Laboratory of Oncology in South China, Collaborative Innovation Center for Cancer Medicine, Guangzhou, 510060 People’s Republic of China; 3 Department of Medical Records, The First Affiliated Hospital, Sun Yat-sen University, Guangzhou, 510060 People’s Republic of China; 4 Yuexiu District Center for Disease Control and Prevention, Guangzhou, 510055 People’s Republic of China; 5 Dadong Street Community Health Service Center, Guangzhou, 510000 People’s Republic of China

**Keywords:** Positive predictive value, Preliminary screening, Screening algorithm, Colorectal cancer

## Abstract

**Background:**

The positive predictive value (PPV) of high risk factor questionnaire (HRFQ) plus fecal immunochemical test (FIT) as preliminary screening strategy for colorectal-related neoplasia is relatively low. We aim to explore independent factors associated with PPVs of HRFQ combined FIT for selecting high risk individuals for colonoscopy.

**Methods:**

A total of 6971 residents were enrolled in a community-based screening program. Participants who had positive results of HRFQ and/or FIT and subsequently received colonoscopy were involved. The associations of socio-demographic factors, lifestyle behaviors, and high risk factors of colorectal cancer with PPVs of HRFQ, FIT, and their combination were evaluated by multivariable logistic regression models.

**Results:**

Among 572 involved cases, 249 (43.5%) colorectal neoplasms were detected by colonoscopy, including 71 advanced adenoma (12.4%) and 9 colorectal cancer (CRC) (1.6%). The PPVs of preliminary screening were 43.5% for total colorectal neoplasms, 14.0% for advanced neoplasm, and 1.6% for CRC. Adding positive HRFQ to FIT could improve the PPV from 3.5 to 8.0% for detecting CRC. Preliminarily screened positive individuals who were males [adjusted odds ratio (AOR): 1.95, 95% CI 1.31, 2.90; *p*  < 0.001], elders (> 60 years) (AOR: 1.70, 95% CI 1.17, 2.46; *p*  = 0.005), or ex-/current smokers (AOR: 3.04, 95% CI 1.31, 7.09; *p * = 0.10) had higher odds of PPVs of detecting colorectal neoplasms.

**Conclusions:**

Combining HRFQ and FIT could largely improve PPVs for screening advanced neoplasm and CRC. Gender and age-specific FIT cut-off values as well as initiating ages for CRC screening might be recommended to improve the accuracy and effectiveness of current screening algorithm.

## Introduction

Colorectal cancer (CRC) is one of the leading causes of cancer death worldwide, with estimating 1.8 million new incident cases and 881,000 death cases in 2018 [1]. In China, CRC is also one of the commonly diagnosed cancers, with its incidence and mortality rate ranking the 4th and 5th of all malignant tumors, respectively [2]. It has observed a steadily increasing trend of CRC incidence and mortality in China in recent decades, along with increasing aging population [3, 4].

There are widespread differences in CRC screening measures, e.g., colonoscopy as gold standard, flexible sigmoidoscopy and stool-based tests such as the faecal occult blood test (FOBT). In China, due to the limited economic resources and health structure and infrastructure, a two-step sequential screening strategy is recommended by China National Commission of Cancer Early Detection and Treatment [5]: eligible individuals are preliminarily screened by a validated high risk factor questionnaire (HRFQ) or fecal immunochemical test (FIT), and positive cases identified in preliminary screening step are further referred for colonoscopy confirmation.

CRC screening has been implemented in several regions of China over the past decade [6, 7]. A preliminary screening using HRFQ along with FIT to identify high-risk individuals of CRC for further confirmation could partly reduce the number of individuals for colonoscopy examination. However, accumulated data shows that preliminary screening of HRFQ plus FIT has a relatively low positive predictive values (PPVs) for selecting high-risk cases of colorectal-related neoplasia [7–10]. These results indicate that individuals with positive result in preliminary screening would have less than 20% probability of colorectal neoplasm and 2% of CRC, respectively. Our data in Guangzhou showed relatively high PPVs for colorectal neoplasms (including polyp, non-advanced and advanced adenoma, and CRC), with 50.4% of HRFQ, 62.1% of FIT, and 58.5% of FIT plus HRFQ, respectively [11]. The low PPVs of preliminary screening suggests that a large proportion of positive individuals identified are actually false positive, leading to unnecessary colonoscopies and extra costs. The false positive results of preliminary screening would increase participants’ discomfort, psychological stress (e.g., anxiety, lowering quality of life), and the risk of complications that could occur during the diagnostic procedure, which further reduce the compliance in subsequent screening programs [12]. In addition, inaccurate preliminary screening results may hinder optimal screening practice. Thus, it is important to depict the potential factors associated with PPVs of preliminary screening for identifying high risk individuals for colonoscopy examination, and such information would be helpful for improving the efficacy and accuracy of a two-stage population-based CRC screening, especially in economically and medically underserved regions.

Epidemiological studies have identified some risk factors of CRC (e.g., gender, older age, smoking, drinking, red meat consumption) [13, 14]. Certain subgroup individuals were observed with higher PPVs in CRC screening program. For instance, the PPV of fecal occult blood test (e.g., FIT) was substantially higher in males and elders [15–18]. However, previous studies compared PPVs in different subpopulations but less likely to delve into multiple factors associated with PPVs based on community-based screening programs. In this study, by using the data from a community-based CRC screening program, we aimed to investigate the potential multiple factors (including socio-demographic factors, risk behaviors, high risk factors of CRC) associated with PPVs of HRFQ combined FIT as preliminary screening strategy to select high-risk individuals for colonoscopy examination in China.

## Methods

### Participants and screening strategy

Participants in this study were a sub-sample from a community-based CRC screening launched in Guangzhou, China of 2014. The eligible participants were community residents aged 50–74 years. A two-step CRC screening strategy was applied. Participants were preliminarily screened by a validated HRFQ or FIT [7], and those with positive HRFQ or FIT results were defined at high-risk of CRC and further referred to colonoscopy confirmation.

**Fig. 1:**
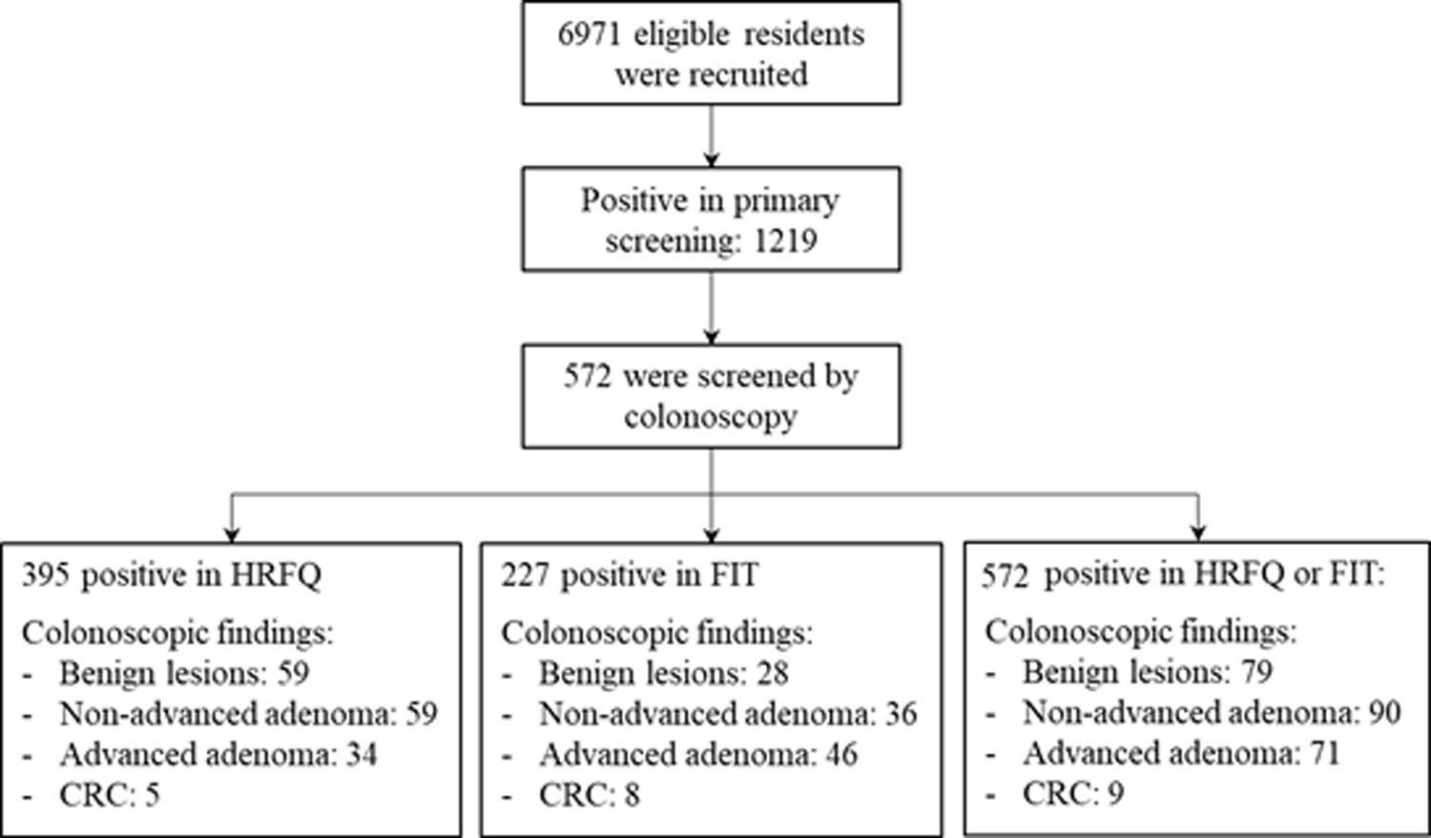
Participants’ flowchart. *HRFQ* high risk factor questionnaire; *FIT* fecal immunological test; *CRC* colorectal cancer

Up to Dec. 2018, a total of 6971 community residents were preliminarily screened. The data of 572 participants, who were with positive test in preliminary screening and subsequently received colonoscopy, was analyzed in this study (see Fig. 1).

### High risk factor questionnaire

All eligible participants were evaluated by HRFQ. Individuals who had one or more of the following risk factors are defined as HRFQ positive: (1) a family history of first-degree relatives with CRC; (2) a personal history of cancers; (3) history of intestinal polyps; or (4) at least two of the following events: chronic diarrhea; chronic constipation; mucous and bloody stool; history of appendicitis or appendectomy; history of chronic cholecystitis or cholecystectomy; history of psychiatric trauma (e.g., divorce, death of relatives) in the past 20 years.

### Fecal immunological tests

Fecal immunological test (FIT) was applied to detect occult blood in stools. Each subject was provided with two collection kits (supplied by ABON, China), and required to collect 5 g stool twice in two consecutive weeks, following the manufacturer’s recommendations, with a detection threshold of 200 ng/mL hemoglobin. Samples were sent to local community health centers within 6 h after collection. All subjects were required to undergo a second-round test, regardless of the result in the first-round test.

### Colonoscopy and histopathological examinations

Participants with positive results in the preliminary screening were defined as high-risk cases of CRC, and referred for further colonoscopy examination. Colonoscopy examination was performed by gastroenterologists in endoscopy units of Sun Yat-sen University Cancer Center (SYSUCC) or authorized medical centers. Each participant who underwent colonoscopy was given a standardized bowel preparation by taking polyethylene glycol (Klean-Prep; HelsinnBirex Pharmaceuticals Ltd., Dublin, Ireland). A withdrawal time of at least 6 min was practiced for all participants, which was according to the current quality indicators for colonoscopy. All colonoscopic findings were documented, including cecal intubation time and the adequacy of bowel preparation. Participants with inadequate bowel preparation would undergo a second round of colonoscopy after an adequate bowel preparation.

Polyps of radius  < 0.5 cm were resected colonoscopically during the examination, if possible. Any neoplasm  ≥ 0.5 cm was biopsied first and proceeded with polypectomy or colectomy depending on pathological report and feasibility of endoscopic surgery. Abnormal findings in colonoscopy included benign lesions (i.e., intestinal polyp, enterelcosis, and non-adenomatous lesions), non-advanced adenoma, advanced adenoma, and CRC. Advanced adenoma was defined as adenoma of  ≥ 10 mm or with a histological examination showing either a 20% or more villous component or high-grade dysplasia. Advanced neoplasm includes advanced adenoma and CRC, while colorectal neoplasms include non-adenomatous benign lesions, non-advanced and advanced adenoma, and CRC.

### Outcome variables and statistical analyses

The primary outcomes included PPVs of HRFQ, FIT and their combination, respectively. Continuous variables (i.e., age, BMI) were described as mean with standard deviation, while categorical variables were described as frequency with percentage. Three multivariable logistic regression models were conducted to investigate the independent factors associated with PPVs of HRFQ, FIT, and their combination, respectively, with false positivity (i.e., a positive result in preliminary screening stage but normal findings in colonoscopy confirmation stage) as reference group. All variables were entered into the final regression models. All analyses were performed by SAS software (version 9.4, SAS Institute). A two-sided *p* value  < 0.05 was considered statistically significant.

### Ethical approval

This study was approved by the institutional review board of SYSUCC, and written informed consents were obtained from all participants. The authenticity of this article has been validated by uploading the key raw data onto the Research Data Deposit (RDD) public platform (www.researchdata.org.cn), with the approval RDD number as RDDA2021122878.

## Results

### Sample characteristics

The mean age of 572 participants was 59.6 years (standard deviation: 7.4 years). Around 61.4% were females, and 73.1% participants had a middle school or below education level. The mean BMI was 23.2 kg/m^2^ (standard deviation: 3.5 kg/m^2^). Around 12.6% participants were overweight or obese, 6.9% had a history of night work, 4.6% had a history of diabetes, and 29.4% were sedentarily more than half time in a typical working day. The proportions of participants with symptoms of chronic constipation, chronic diarrhea, and hematochezia were 25.7%, 24.0%, and 25.9%, respectively (Table 1).

**Table 1:** Description of sample characteristics

	Positive in primary screening			Abnormal in colonoscopy (*n* = 249)
	Total (*n* = 572)	HRFQ + (*n* = 395)	FIT + (*n* = 227)	
Gender, *n* (%)				
Male	221 (38.6)	148 (37.5)	94 (41.4)	126 (50.6)
Female	351 (61.4)	247 (62.5)	133 (58.6)	123 (49.4)
Age, years, mean ± SD	59.6 ± 7.4	59.0 ± 7.2	60.5 ± 7.9	61.2 ± 7.3
≤ 60	308 (53.8)	227 (57.5)	104 (45.8)	112 (45.0)
> 60	264 (46.2)	168 (42.5)	123 (54.2)	137 (55.0)
Education level, *n* (%)				
Primary school or below	39 (6.8)	24 (6.1)	218 (7.9)	23 (9.2)
Middle school	379 (66.3)	251 (63.5)	154 (67.8)	163 (65.5)
College or above	154 (26.9)	120 (30.4)	55 (24.2)	63 (25.3)
Ex-smoker/current smoker, *n* (%)	47 (8.2)	32 (8.1)	19 (8.4)	36 (14.5)
Alcohol drinking, *n* (%)	16 (2.8)	13 (3.3)	3 (1.3)	11 (4.4)
History of night work, *n* (%)	39 (6.9)	32 (8.1)	9 (4.0)	16 (6.4)
Sedentary more than half in work time, *n* (%)	168 (29.4)	117 (29.6)	74 (32.6)	83 (33.3)
History of diabetes, *n* (%)	26 (4.6)	20 (5.1)	10 (4.4)	12 (4.8)
BMI, kg/m^2^, mean ± SD	23.2 ± 3.5	23.2 ± 3.5	23.4 ± 4.0	23.5 ± 3.9
Overweight or obesity, *n* (%)	72 (12.6)	52 (13.2)	27 (11.9)	37 (14.9)
High risk factors*, n* (%)				
First degree relatives with CRC	113 (19.8)	113 (28.6)	15 (6.6)	48 (19.3)
History of cancer	29 (5.1)	29 (7.3)	6 (2.6)	11 (4.4)
History of polypus	104 (18.2)	104 (26.3)	13 (5.7)	46 (18.5)
Chronic constipation	147 (25.7)	133 (33.7)	37 (16.3)	52 (20.9)
Chronic diarrhea	137 (24.0)	124 (31.4)	29 (12.8)	53 (21.3)
Hematochezia	148 (25.9)	142 (35.9)	29 (12.8)	60 (24.1)
Chronic appendicitis	61 (10.7)	54 (13.7)	9 (4.0)	27 (10.8)
Chronic cholecystitis	44 (7.7)	41 (10.4)	7 (3.1)	15 (6.0)
Psychiatric trauma in the past 20 years	68 (11.9)	67 (17.0)	11 (4.9)	23 (9.2)

*FIT* fecal immunological test; *SD* standard deviation; *BMI* body mass index; *CRC* colorectal cancer

### Summary of screening performance

Among 572 preliminarily positive individuals, 395 participants (69.1%) were identified with a positive HRFQ, 227 (39.7%) with a positive FIT, and 50 (8.7%) with both positive HRFQ and FIT. A total of 249 (43.5%) colorectal neoplasms were detected in colonoscopy examination, including 79 non-adenomatous benign lesions (13.8%), 90 non-advanced adenoma (15.7%), 71 advanced adenoma (12.4%), and 9 CRC (1.6%) (Fig. 1; Table 2).

**Table 2:** Results of the two-step screening strategy

Primary screening	Total	Colorectal neoplasms		Advanced neoplasm		CRC	
		*n*	PPV (95% CI)	*n*	PPV (95% CI)	*n*	PPV (95% CI)
HRFQ positive	395	157	39.7 (34.9, 44.8)	39	9.9 (7.1, 13.3)	5	1.3 (0.4, 2.9)
FIT positive	227	118	52.0 (45.3, 58.6)	54	23.8 (18.4, 30.0)	8	3.5 (2.8, 12.0)
HRFQ or FIT	572	249	43.5 (39.4, 47.7)	80	14.0 (11.3, 17.1)	9	1.6 (0.8, 3.1)
Only HRFQ positive	345	131	38.0 (32.8, 43.3)	26	7.5 (5.0, 10.9)	1	0.3 (0.01, 1.6)
Only FIT positive	177	92	52.0 (44.4, 59.5)	41	23.2 (17.2, 30.1)	4	2.3 (0.6, 5.7)
HRFQ and FIT positive	50	26	52.0 (37.4, 66.3)	13	26.0 (14.6, 40.3)	4	8.0 (2.2, 19.2)

Colorectal neoplasms include non-adenomatous benign lesions, non-advanced adenoma, advanced adenoma, and colorectal cancer

Advanced neoplasm includes advanced adenoma and colorectal cancer

*HRFQ* high risk factor questionnaire; *FIT* fecal immunological test; *PPV* positive predictive value; *CRC* colorectal cancer

The overall PPVs of preliminary screening strategy were 43.5% (95% CI 39.4, 47.7) for colorectal neoplasms, 14.0% (95% CI 11.3, 17.1) for advanced neoplasm, and 1.6% (95% CI 0.8, 3.1) for CRC. The PPVs of HRFQ were 39.7% (95% CI 34.9, 44.8) for colorectal neoplasms, 9.9% (95% CI 7.1, 13.3) for advanced neoplasm, and 1.3% (95% CI 0.4, 2.9) for CRC, while the values of FIT were 52.0% (95% CI 45.3, 58.6), 23.8% (95% CI 18.4, 30.0), and 3.5% (95% CI 2.8, 12.0), respectively. Positive results with both HRFQ and FIT had the highest PPVs for advanced neoplasm (26.0%, 95% CI 14.6, 40.3) and CRC (8.0%, 95% CI 2.2, 19.2) (Table 2).

### Factors associated with PPVs of preliminary screening for colorectal neoplasms

Among 572 participants with positive results in preliminary screening, individuals who were males [adjusted odd ratio (AOR): 1.95, 95% CI 1.31, 2.90; *p*  < 0.001], older than 60 years (AOR: 1.70, 95% CI 1.17, 2.46; *p*  = 0.005), or ex-smokers/current smokers (AOR: 3.04, 95% CI 1.31, 7.09; *p*  = 0.10) had increased odds of PPVs (Table 3). For 395 participants with positive HRFQ, males (AOR  = 1.79, 95% CI 1.10, 2.90; *p*  = 0.018) or ex-smokers/current smokers (AOR: 3.04, 95% CI 1.14, 8.11; *p*  = 0.027) were significantly associated with higher odds of PPVs (Table 3). For those with positive FIT, males (AOR: 2.50, 95% CI 1.31, 4.77; *p*  = 0.006) or older than 60 years (AOR: 2.06, 95% CI 1.11, 3.84; *p*  = 0.023) were more likely to have higher PPVs (Table 3). We did not find other factors those were significantly associated with PPVs in positive HRFQ, FIT or their combination (Table 3).

**Table 3:** Factors associated with positive predictive values of HRFQ and FIT as preliminary screening for colorectal neoplasms

	HRFQ positive (*n* = 395)		FIT positive (*n* = 227)		HRFQ or FIT positive (*n* = 572)	
	PPV, *n* (%)	AOR (95% CI)	PPV, *n* (%)	AOR (95% CI)	PPV, *n* (%)	AOR (95% CI)
Gender						
Female	80 (32.4)	**1**	54 (40.6)	**1**	123 (35.0)	**1**
Male	77 (52.0)	1.79 (1.10, 2.90)*	64 (68.1)	2.50 (1.31, 4.77)**	126 (57.1)	1.95 (1.31, 2.90)***
Age, years						
≤ 60	81 (35.7)	1	41 (39.4)	**1**	112 (36.4)	**1**
> 60	76 (45.2)	1.47 (0.93, 2.31)	77 (62.6)	2.06 (1.11, 3.84)*	137 (51.9)	1.70 (1.17, 2.46)**
Education level						
Primary school or below	12 (50.0)	1	13 (72.2)	1	23 (59.0)	1
Middle school	94 (37.5)	0.79 (0.32, 2.16)	83 (53.9)	0.58 (0.17, 1.97)	163 (43.0)	0.66 (0.32, 1.34)
College or above	51 (42.5)	0.83 (0.32, 2.16)	22 (40.0)	0.28 (0.07, 1.09)^†^	63 (40.9)	0.56 (0.26, 1.21)
Smoking						
No	64 (36.4)	**1**	38 (46.9)	1	91 (39.2)	**1**
Ex-smoker/current smoker	23 (71.9)	3.04 (1.14, 8.11)*	17 (89.5)	2.94 (0.54, 16.02)	36 (76.6)	3.04 (1.31, 7.09)*
Alcohol drinking						
No	79 (40.5)	1	52 (53.6)	–	116 (44.1)	1
Yes	8 (61.5)	1.26 (0.30, 5.23)	3 (100.0)		11 (68.8)	1.62 (0.44, 5.99)
History of night work						
No	77 (45.3)	1	51 (55.4)	1	114 (48.5)	1
Yes	11 (34.4)	0.71 (0.29, 1.74)	6 (66.7)	0.95 (0.12, 7.48)	16 (41.0)	0.80 (0.36, 1.78)
Sedentary more than half time in work						
No	34 (40.0)	1	13 (50.0)	1	45 (42.9)	1
Yes	53 (45.3)	1.05 (0.55, 1.99)	43 (58.1)	1.77 (0.58, 5.38)	83 (49.4)	1.19 (0.68, 2.07)
History of diabetes						
No	149 (39.7)	1	110 (50.7)	1	237 (43.4)	1
Yes	8 (40.0)	0.78 (0.28, 2.17)	8 (80.0)	2.16 (0.33, 14.52)	12 (46.2)	0.72 (0.29, 1.77)
BMI, kg/m^2^						
< 25	64 (41.8)	1	35 (50.0)	1	89 (44.1)	1
≥ 25	23 (44.2)	0.86 (0.42, 1.74)	19 (70.4)	1.48 (0.46, 4.80)	37 (51.4)	1.02 (0.56, 1.87)
High risk factors						
First degree relatives with CRC						
No	109 (38.7)	1	110 (51.9)	1	201 (43.8)	1
Yes	48 (42.5)	1.14 (0.63, 2.05)	8 (53.3)	1.39 (0.40, 4.87)	48 (42.5)	0.96 (0.60, 1.53)
History of cancer						
No	146 (39.9)	1	115 (52.0)	1	238 (43.8)	1
Yes	11 (37.9)	1.07 (0.45, 2.58)	3 (50.0)	1.77 (0.22, 14.40)	11 (37.9)	0.89 (0.39, 2.01)
History of polypus						
No	111 (38.1)	1	113 (52.8)	1	203 (43.4)	1
Yes	46 (44.2)	1.12 (0.64, 1.96)	5 (38.5)	0.89 (0.22, 3.51)	46 (44.2)	0.96 (0.60, 1.52)
Chronic constipation						
No	111 (42.4)	1	103 (54.2)	1	197 (46.4)	1
Yes	46 (34.6)	0.79 (0.48, 1.31)	15 (40.5)	0.61 (0.25, 1.45)	52 (35.4)	0.69 (0.45, 1.07)^†^
Chronic diarrhea						
No	108 (39.9)	1	105 (53.0)	1	196 (45.1)	1
Yes	49 (39.5)	1.04 (0.63, 1.71)	13 (44.8)	0.88 (0.35, 2.22)	53 (38.7)	0.86 (0.55, 1.33)
Hematochezia						
No	101 (39.9)	1	103 (52.0)	1	189 (44.6)	1
Yes	56 (39.4)	1.05 (0.63, 1.72)	15 (51.7)	2.15 (0.76, 6.11)	60 (40.5)	0.98 (0.63, 1.51)
Chronic appendicitis						
No	135 (39.6)	1	111 (50.9)	1	222 (43.4)	1
Yes	22 (40.7)	1.12 (0.59, 2.10)	7 (77.8)	3.68 (0.57, 23.07)	27 (44.3)	1.11 (0.63, 1.96)
Chronic cholecystitis						
No	143 (40.4)	1	116 (52.7)	1	234 (44.3)	1
Yes	14 (34.2)	0.68 (0.32, 1.44)	2 (28.6)	0.50 (0.07, 3.39)	15 (34.1)	0.61 (0.30, 1.22)
Negative life events in the past 20 years						
No	135 (41.2)	1	114 (52.8)	1	226 (44.8)	1
Yes	22 (32.8)	0.72 (0.39, 1.33)	4 (36.4)	0.25 (0.05, 1.41)	23 (33.8)	0.73 (0.41, 1.32)

Values in bold indicate *p*  < 0.05

*HRFQ* high risk factor questionnaire; *FIT* fecal immunological test; *PPV* positive predictive value; *BMI* body mass index; *CRC* colorectal cancer; *AOR* adjusted odds ratio; *95% CI* 95% confidence interval

^†^*p*  < 0.10; *p  < 0.05; **p  < 0.01; ***p  < 0.001

## Discussion

In this community-based CRC screening program, we mainly evaluated the screening performance and associated factors with PPVs of preliminary screening for the detection of colorectal neoplasia. It is observed that the PPVs of preliminary screening were 43.5% for colorectal neoplasms, 14.0% for advanced neoplasm, and 1.6% for CRC. The PPVs of FIT were substantially higher than those of HRFQ for screening colorectal neoplasms, advanced neoplasm, and CRC, respectively. Moreover, the addition of positive HRFQ to FIT could largely improve PPV from 3.5 to 8.0% for the detection of CRC. In addition, our findings showed that preliminarily screened positive individuals who were males, elderly individuals, or ex-smokers/current smokers had significantly increased probability to be detected with colorectal neoplasia (i.e., colorectal neoplasms, advanced neoplasm, or CRC), and should be highly recommended to colonoscopy examination.

Consistent with previous reports[15, 18–20], our findings indicated sex- and age-specific disparities on PPVs of HRFQ and FIT as preliminary screening strategy in detecting colorectal neoplasms. Higher PPVs in these subpopulations could be partially explained by their higher CRC prevalence. It is well-known that the prevalence of advanced colorectal neoplasms was higher in males than that in females [7, 21], and significantly increased with age [18, 22]. Similarly, an age gradient of PPV for colorectal neoplasms detection was observed, which was in line with the increased CRC incidence with age [20, 23]. Anatomically, females have longer colonic length and transit time than males [24, 25], leading to more degradation of hemoglobin before defecation, lower fecal hemoglobin concentration, and decreased chance to test a positive result of fecal occult blood by FIT. Longer colonic length and prior abdominal pelvic surgeries (e.g., caesarean section) also pose difficulties for colonoscopists [26]. Besides, females have higher proportion of right-sided carcinoma than males, and it is therefore less likely to detect bleeding caused by colorectal neoplasm [27].

Apart from abovementioned anatomical and physiological disparities, factors associated with health behaviors and beliefs might also contribute to gender discrepancies in PPVs of preliminary screening for colorectal neoplasia. Males who conformed to masculinity norms (i.e., self-reliance, avoidance of femininity, heterosexual self-presentation, and risk-taking) might be motivated by more severe symptoms to participate in cancer screening and present superior PPVs [28–30]. In addition, females reported significant discomfort during colonoscopy [31], and had lower completion rate of colonoscopy [32], as compared with males. Therefore, colorectal neoplasms might be missed by the “gold standard”, resulting in lower PPVs in females. Abovementioned factors might play a synergistic role in screening and eventually cause sex-specific disparities of the PPV of FIT.

Our findings further showed that ex-smokers/current smokers with positive preliminary results had higher PPVs, and had increased probability to be detected with colorectal neoplasia by colonoscopy. Smoking has been confirmed as a significant risk factor of CRC due to the carcinogenicity of nicotine [33]. The evidences from meta-analyses revealed that smoking would significantly increase the incidence of colorectal polyps and CRC, with dose–response relationships [34–36]. High incidence of CRC in smokers might partially explain the higher PPV of preliminary screening for colorectal neoplasia in this subpopulation. In addition, it is observed that smokers were more likely to be accompanied with other high-risk behaviors (e.g., alcohol drinking, low physical activity, intake of red meat) [37, 38], indicating that smokers might be more likely exposed to multiple risk factors of CRC, eventually with higher PPVs in cancer screening compared to nonsmokers. Besides, smokers who underwent screening might simultaneously be motivated by other non-negligible CRC-related symptoms, therefore presenting superior PPVs of colorectal neoplasms.

Our findings have several significant implications. First, it suggests that males, elders (age  > 60) and smokers who were identified as high-risk individuals of colorectal neoplasms in the preliminary screening stage should be given priority to refer for colonoscopy. These subpopulations, usually presenting low screening rate of CRC [39, 40], need to be paid extra effort to raise their awareness and compliance for colonoscopy screening. Second, age, gender and smoking were just qualitatively incorporated in some risk scoring systems for CRC screening [41–43] (e.g., Asia–Pacific Colorectal Screening score). Mathematical algorithm quantitatively weighting these factors might improve the screening accuracy and efficacy to select asymptomatic participants eligible for colonoscopy in the future. Thirdly, it might be more effective and flexible to design individualized preliminary screening strategy based on personal risk level of colorectal neoplasia. Age and gender-specific cut-off values of FIT could improve the performance of FIT in CRC screening [44, 45]. Meanwhile, based on the evidence that CRC occurs earlier in smokers than non-smokers [46, 47], and the higher life expectancy in females versus males and non-smokers versus smokers, the optimal initiating age for CRC screening is suggested to be 5–10 years later for females than males, while 5–10 years earlier for smokers than non-smokers among general population [48, 49]. Therefore, individualized cut-off values of FIT and initiating screening age by gender and smoking status might be novel screening scheme to reduce the false positive rate and improve the cost-effectiveness of FIT-based screening in the future.

However, there are some limitations that should be addressed when interpreting our findings. First, the small sample size might cause bias. For instance, smoking was not associated with higher PPV in the FIT-positive population, which might be partially due to the small sample of this subgroup (*n*  = 17). Small sample size also poses difficulty in conducting potential stratified analyses, such as dose–response relationship between smoking and PPVs of preliminary screening. Besides, we only investigated one district of Guangzhou in China. Therefore, the generalizability of our findings should be cautious, and be further validated in large-scale representative samples. Second, considering that HRFQ was a self-reporting tool, participants may misestimate some risk factors. People who underwent colonoscopy were obviously more inclined to receive screening and they might exaggerate their symptoms, leading to an increased false-positive rate. Third, it did not involve other potential epidemiological factors (e.g., history of NSAIDS medication, calcium taking, and diet habits) in the analyses due to the restriction of the study database. Several female-specific factors (e.g., menopause and history of gynecology tumor in early age) also deserve considerations as they were reported to be associated with the risk of adenomas or CRC [50, 51]. In addition, we did not classify the colorectal neoplasm based on clinical characteristics (e.g., neoplasm site). In the past decades, the incidence of rectal carcinoma has increased more significantly than colon carcinoma, indicating that these two kinds of carcinoma might have more discrepancies to be investigated [52]. For those risk factors involved in this study, it would be more definite and meaningful to set more categories in each variable. Fourth, with the application of HRFQ, our study actually recruited a mix of symptomatic and asymptomatic individuals, which might partially explain the higher PPVs of preliminary screening as compared to those reported in the previous reports. Fifth, as participants with negative results in the preliminary screening were not routinely required to receive colonoscopy per protocol, the negative predictive values of preliminary screening were not calculated and reported.

In sum, our findings showed that a preliminary screening by combining HRFQ and FIT could largely improve PPVs for screening advanced neoplasm and CRC compared to HRFQ- or FIT-alone strategy. Males, elderly individuals, and smokers were associated with higher PPVs in a two-step screening strategy. These subpopulations might be the prime target of propaganda. Individualized FIT cut-off values and initiating ages by gender, age and smoking status for screening might be an attractive option to improve the accuracy of current screening algorithm.

## Data Availability

The datasets generated during and/or analysed during the current study are available in the RDD public platform (www.researchdata.org.cn), with the approval RDD number as RDDA2021122878.
